# Production of Extracellular Traps against *Aspergillus fumigatus In Vitro* and in Infected Lung Tissue Is Dependent on Invading Neutrophils and Influenced by Hydrophobin RodA

**DOI:** 10.1371/journal.ppat.1000873

**Published:** 2010-04-29

**Authors:** Sandra Bruns, Olaf Kniemeyer, Mike Hasenberg, Vishukumar Aimanianda, Sandor Nietzsche, Andreas Thywißen, Andreas Jeron, Jean-Paul Latgé, Axel A. Brakhage, Matthias Gunzer

**Affiliations:** 1 Department of Molecular and Applied Microbiology, Leibniz Institute for Natural Product Research and Infection Biology - Hans-Knoell-Institute (HKI), Jena, Germany; 2 Department of Microbiology and Molecular Biology, Friedrich Schiller University Jena, Jena, Germany; 3 Otto-von-Guericke University Magdeburg, Institute for Molecular and Clinical Immunology, Magdeburg, Germany; 4 Unite des Aspergillus, Institut Pasteur, Paris, France; 5 Electron Microscopic Centre, Clinics of the Friedrich Schiller University Jena, Jena, Germany; 6 Helmholtz-Institute for Infection Research (HZI), Braunschweig, Germany; David Geffen School of Medicine at University of California Los Angeles, United States of America

## Abstract

*Aspergillus fumigatus* is the most important airborne fungal pathogen causing life-threatening infections in immunocompromised patients. Macrophages and neutrophils are known to kill conidia, whereas hyphae are killed mainly by neutrophils. Since hyphae are too large to be engulfed, neutrophils possess an array of extracellular killing mechanisms including the formation of neutrophil extracellular traps (NETs) consisting of nuclear DNA decorated with fungicidal proteins. However, until now NET formation in response to *A. fumigatus* has only been demonstrated *in vitro*, the importance of neutrophils for their production *in vivo* is unclear and the molecular mechanisms of the fungus to defend against NET formation are unknown. Here, we show that human neutrophils produce NETs in *vitro* when encountering *A. fumigatus*. In time-lapse movies NET production was a highly dynamic process which, however, was only exhibited by a sub-population of cells. NETosis was maximal against hyphae, but reduced against resting and swollen conidia. In a newly developed mouse model we could then demonstrate the existence and measure the kinetics of NET formation *in vivo* by 2-photon microscopy of *Aspergillus*-infected lungs. We also observed the enormous dynamics of neutrophils within the lung and their ability to interact with and phagocytose fungal elements *in situ*. Furthermore, systemic neutrophil depletion in mice almost completely inhibited NET formation in lungs, thus directly linking the immigration of neutrophils with NET formation *in vivo*. By using fungal mutants and purified proteins we demonstrate that hydrophobin RodA, a surface protein making conidia immunologically inert, led to reduced NET formation of neutrophils encountering *Aspergillus* fungal elements. NET-dependent killing of *Aspergillus*-hyphae could be demonstrated at later time-points, but was only moderate. Thus, these data establish that NET formation occurs *in vivo* during host defence against *A. fumigatus*, but suggest that it does not play a major role in killing this fungus. Instead, NETs may have a fungistatic effect and may prevent further spreading.

## Author Summary

The fungus *Aspergillus fumigatus* grows on decaying organic matter and produces large numbers of spores, called conidia, which are constantly inhaled by humans. This is harmless, because we have a functioning defence system of immune cells called neutrophil granulocytes, but people with too few or non-functioning neutrophils can die of *Aspergillus* infections. Neutrophils invade the lung, engulf/phagocytose and thereby kill conidia. Dying neutrophils can also throw their nuclear DNA on hyphal elements as NETs (Neutrophil Extracellular Traps) that are decorated with antimicrobial proteins. Thus, larger fungal amounts, including tissue-invading hyphae, can still be controlled. However, until today the formation of NETs has not been demonstrated in *Aspergillus*-infected lungs, the role of neutrophils for this process was unknown and whether the fungus has anti-NET defence strategies on its own was not clear. We demonstrate here the existence of NETs in *Aspergillus*-infected lungs, show that neutrophils produce these structures and that they phagocytose fungal elements within the lung tissue. Furthermore, we show that *Aspergillus* camouflages its spores by means of the surface protein hydrophobin RodA, which is able to strongly prevent NET formation by neutrophils. These studies shed new light on the dynamics and molecular mechanisms of this key process of host-pathogen interaction. Although these data establish that NET formation occurs *in vivo* during host defence against *A. fumigatus*, we suggest that NET formation does not play a major role in killing this fungus.

## Introduction

*Aspergillus fumigatus* is the most important airborne fungal pathogen causing life-threatening infections in immunocompromised patients. Conidia, the asexually produced small fungal spores, are inhaled and reach the lung alveoli, where they are confronted with the first line of defence which is built up of resident alveolar macrophages and newly recruited neutrophil granulocytes (neutrophils). Conidia are thought to be killed by macrophages whereas hyphae are mainly attacked by neutrophils (reviewed in: [1]–[3]). However, recruited neutrophils are also able to phagocytose conidia directly [4], [5] or prevent their germination as shown by Bonnett et al. [6]. Furthermore, the essential role of neutrophils in preventing invasive growth of *A. fumigatus* has recently been proven [7]. Nevertheless, the detailed mechanisms how these immune effector cells protect the human host against *A. fumigatus* are still a matter of debate.

The NAD(P)H oxidase in phagocytes is regarded to be essential for host defence against aspergillosis. This idea is supported by the fact that patients with chronic granulomatous disease are highly susceptible to fungal diseases, especially *Aspergillus* infections. Neutrophils of these patients show markedly deficient NAD(P)H oxidase activity [8]. The activation of NAD(P)H oxidase results in the formation of superoxide anions and other reactive oxygen intermediates (ROI) (reviewed in [1]). However although the catalase or yap1 and skn7 mutants display an increased sensitivity to ROIs *in vitro*, these detoxifying systems of *A. fumigatus* do not play any role in controlling the killing of *A. fumigatus* conidia by phagocytes *in vivo* [9].

Consequently, the production of ROI by the host may be important for control of *Aspergillus* on a level distinct from direct killing. This result is in agreement with recent findings that the granule proteins in neutrophils are primarily responsible for the killing process of microbes while ROI only function by activating vacuolar enzymes [10], [11]. The contribution of NAD(P)H oxidase in killing conidia in macrophages, as shown for *A. fumigatus* [12], may be indirect by depolarising the phagocytic vacuole, leading to an influx of ions which results in the activation of digestion enzymes, as proposed by Segal [11]. The importance of vacuolar enzymes for fungal defence is also supported by the finding that knock-out mice lacking cathepsin G and elastase were found to be susceptible to *Aspergillus* infection [13].

Hence, the mechanism how the innate immune systems effectively counteracts spores and germlings of *A. fumigatus* has to be further elucidated with a focus on killing mechanisms independent of direct ROI-mediated destruction. In the light of these observations the identification of extracellular fibres called neutrophil extracellular traps (NETs), which are produced as a final act of defence by dying neutrophils may be of major importance [14]. NETs are composed of chromatin covered with granular proteins which express antimicrobial activity. The process of NET formation depends on the induction of a ROI-mediated signaling cascade in neutrophils that ends up in the disintegration of the nuclear envelope and granular membranes [15]. After membrane rupture the NETs are formed by intracellular mixture of nuclear DNA with granular contents and then explosively released in a matter of seconds, a process that is associated with cell death. This unique sequence of events is also called NETosis (reviewed in [16]). NETs may mediate the trapping of conidia of *A. fumigatus* [17], as it has been shown for the yeast form and hyphal cells of *C. albicans* [18] and for *A. nidulans* [19].

Although NETs are an attractive model to explain defence against *A. fumigatus*, direct proof of their existence and importance *in vivo* is still lacking. The restoration of NAD(P)H oxidase activity in hematopoietic stem cells of a human CGD patient by gene transfer has been shown to re-establish NETosis in neutrophils derived from these cells *in vitro* and restore fungal defence against *A. nidulans* in the treated patient. However, the re-establishment of NET formation in this patient *in vivo* as basis for successful fungal defence could not be demonstrated directly and thus remained a matter of speculation [19]. Furthermore, the importance of NETosis for the defence against the clinically much more relevant *A. fumigatus* still lacks experimental proof. Finally, molecular determinants of fungal pathogens that control or induce the production of NETs by binding neutrophils are so far entirely unknown.

To get a better understanding of these issues, we set out to comprehensively study whether the different morphotypes of *A. fumigatus* have the potential to induce NETs *in vitro*. Furthermore, we aimed at shedding light on molecular mechanisms involved. Finally we wanted to clarify, whether NETs are really formed in *Aspergillus*-infected lungs and whether this is dependent on newly arriving neutrophils.

## Results

### Human neutrophils produce extracellular traps when encountering different *A. fumigatus* morphotypes

**Figure 1:**
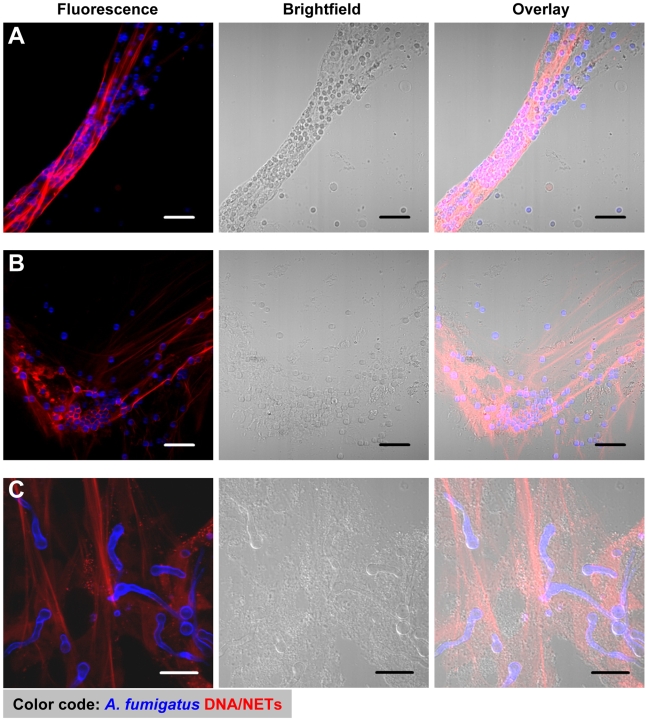
NET formation by human neutrophils co-incubated with resting and swollen conidia or hyphae of *A. fumigatus*. CLSM fluorescence, bright field and overlay images showing NET formation of human neutrophils after co-incubation with *A. fumigatus*. Extracellular DNA was stained with propidium iodide (red), conidia and hyphae with calcofluor white (blue). Microscopic pictures were taken after 3 hours. Neutrophils were co-incubated with resting conidia (A), swollen conidia (B) and hyphae (C). All scale bars represent 20 µm length.

To analyse whether *A. fumigatus* induced the production of NETs by human neutrophils, different morphotypes of *A. fumigatus*, i.e. resting or swollen conidia and hyphae, were co-incubated with human neutrophils for different time periods. Confocal images of cultures stained with propidium iodide and calcofluor white during co-incubation showed, that freshly isolated non-prestimulated neutrophils produced typical NET structures against all morphotypes within three hours (Figure 1 and Figure S1). NET formation started with a rapid enlargement of the neutrophils followed by their final burst. NET formation was visible after 120 min and increased during the following hour of co-incubation (Figure 1, 3A and Video S1). Activation of neutrophils *in vitro* using phorbol-12-myristate-13-acetate (PMA) enhanced this effect (data not shown). Neutrophils alone without fungi or during co-incubation with latex beads did not produce NETs (Figure S1).

**Figure 2:**
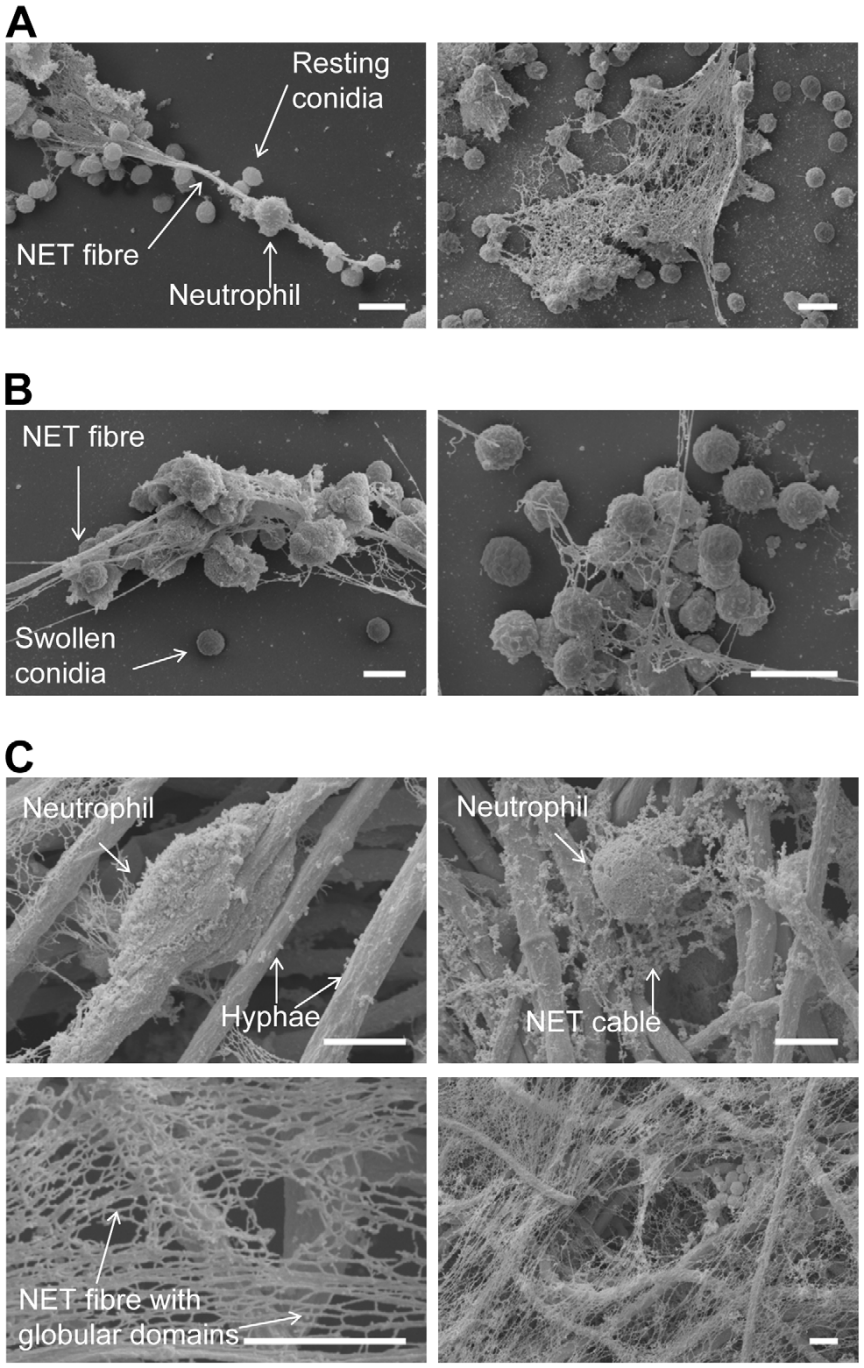
Scanning electron microscopy (SEM) micrographs of conidia and hyphae trapped in NETs. NET formation of human neutrophils after co-incubation with *A. fumigatus*. Microscopic pictures were taken after 3 hours. Neutrophils were co-incubated with resting conidia (A), swollen conidia (B) and hyphae (C). All scale bars represent 5 µm length. Morphological structures are indicated by labelled arrows.

Scanning electron microscopy further revealed the intimate contact between neutrophils and the three morphotypes (Figure 2). Furthermore, it showed the formation of typical NET structures with the different morphological characteristics defined by Brinkmann and Zychlinsky [16], i.e., cables, threads and globular domains (Figure 2C3). The architecture of NETs was thus similar to that seen for NETs induced by other pathogens like *Shigella flexneri* [19]. This suggested, that the overall architecture of NETs is fixed, irrespective of the pathogenic microorganism which was encountered by neutrophils.

**Figure 3:**
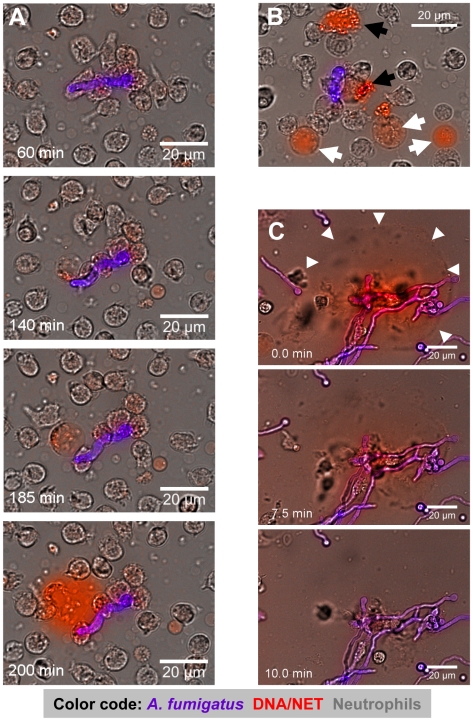
Time-lapse widefield microscopy of NET formation by neutrophils co-incubated with swollen conidia of *A. fumigatus*. (A) Time series of NET formation by human neutrophils upon contact to swollen *A. fumigatus* conidia. Extracellular DNA was stained with propidium iodide (red), conidia and germ tubes with calcofluor white (blue-pink). Microscopy was carried out for 5 h after onset of co-incubation while single pictures were taken every 30 seconds (Video S1). (B) Co-incubation after 150 minutes. Black arrowheads indicate cells which had died without releasing DNA, white arrowheads point to cells undergoing preparation for NET release. The colours are the same as in (A). (C) DNase digestion of NETs after 180 minutes of co-incubation. White arrowheads indicate the preformed NET structures right before destruction by the enzyme (Video S2). The colours are the same as in (A). Where appropriate, real time is indicated in minutes. The DNase was added 7 min before onset of the visible NET digestion to the border of the microscopy chamber right before sealing. The size of scale bars is indicated directly. These movies are representative for at least 6 independent experiments that were performed.

Since the static images did not reveal the cell movements and fungal contacts of neutrophils before final NET formation, we also investigated the process by live cell fluorescence imaging. These analyses allowed to precisely reconstruct the kinetics of the reaction (Figure 3A and Video S1). Normally, neutrophils rapidly phagocytosed conidia, as described [4]. Interestingly, the large hyphal structures, that could not be internalised, were covered and ensheathed by multiple neutrophils. The rate of NET production was dependent on the chosen ratio between neutrophils and fungal elements (data not shown). At a ratio of 1∶1 only a minority of cells in a population (12.4±9.5%) were finally observed to disintegrate and undergo NETosis, which was clearly visible by rapid staining of externalised DNA by the nucleic acid dye propidium iodide in the supernatant (Figure 3A and Video S1). Thus, NETosis was not an invariant response pathway of dying neutrophils and its frequency was further influenced by the E/T ratio. Often only a sub-fraction of neutrophils was able to generate NETs, while the majority of cells remained alive. Nevertheless, even when only few cells were observed to undergo NETosis these could produce NETs of considerable size. A minority of cells died without signs of NET formation (data not shown). The latter was evident from bright red nuclear staining of condensed cells (Figure 3B, black arrows) or swollen cells with dilute cytoplasmic staining (Figure 3B, white arrows).

To further confirm that NETs produced against *A. fumigatus* consisted of DNA, we added DNase I to neutrophil-*Aspergillus* co-cultures containing prominent NET-structures (Figure 3C and Video S2). Within minutes after addition of DNase I NETs were completely disrupted, indicating that NETs observed in these systems were indeed composed of DNA (Figure 3C and Video S2).

### Phagocytosis and NET production by invading neutrophils in the lungs of *Aspergillus*-infected mice

**Figure 4:**
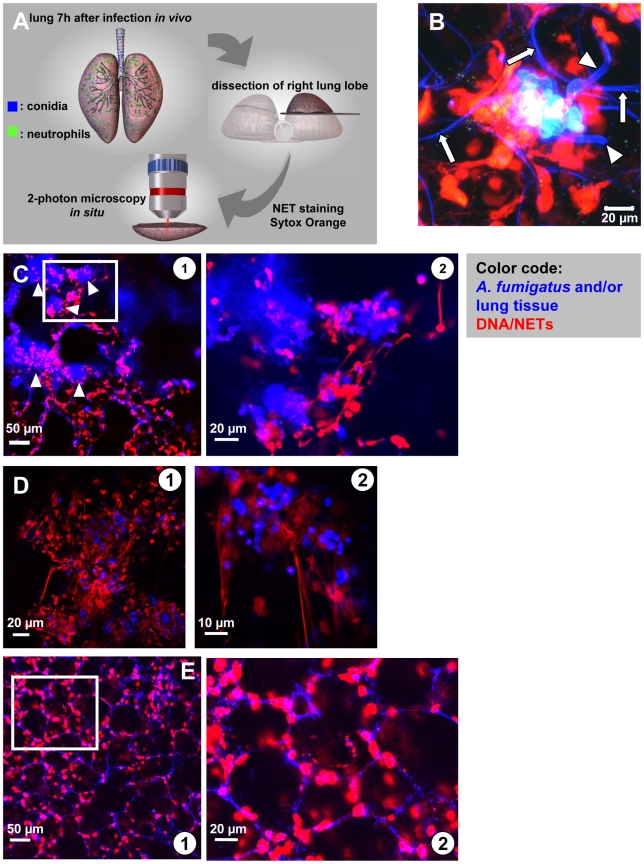
*In situ* 2-Photon microscopy of NET-like structures formed in a murine *A. fumigatus* infection model. (A) Model system used to demonstrate NET formation and -structure in living lung-slices. 7–10 hours after infection of live mice the right lung lobe was prepared, dissected and NETs were stained with a specific DNA dye. *In situ* 2-photon microscopy was carried out in PBS pre-warmed to 37°C (Video S3). (B) High resolution image of a fungal mass with outgrowing hyphae (blue colour, arrowheads) within the infected lung. Red staining (DNA) shows NET-structures as well as the intact nuclei of host cells within the lung. Please also note the fine blue curvature of alveoli (white arrows). The same image is 3-D rendered in Video S8. (C) 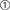 In lungs of infected mice multiple of such large accumulations of fungal masses were visible (blue colour, white arrowheads). At higher magnification 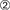 these fungal masses were surrounded by fine, red fibres demonstrating NET formation in these areas (D) In low 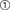 and especially high magnification 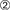 such structures often strongly resembled NETs observed before *in vitro* (red) and were mostly associated with swollen *A. fumigatus* conidia (blue) in lung slices freshly prepared from infected lungs (“acute lung slices”). (E) In mice treated i.t. with PBS NET formation was absent (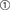 overview, 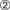 higher magnification). Blue: SHG signal of the lung tissue and fungal masses, red: nuclei of cells cut open during processing. The images are representative of more than 20 individual mice, which were analysed.

The data above suggested, that contact to *A. fumigatus* elements, especially growing hyphae, triggered NET formation by human neutrophils, as previously described for conidia alone [17]. However, like with the study by Jaillon [17] this observation was purely based on *in vitro* experiments. Thus, although NET-structures have been observed in tissue wounds *in vivo* before [14], [20] or in lungs infected with *Candida albicans* [21], it is not known, whether they also exist in lungs recently infected with *A. fumigatus* and also the kinetics of NET formation *in vivo* has not been characterised, yet [19]. As direct imaging of NETs in the lungs of humans is not possible [19], we newly developed a mouse model of early invasive aspergillosis (Figure 4A, and Video S3) allowing us to clarify, whether NETs really occur during defence against an acute *Aspergillus* infection *in vivo*.

We intratracheally injected swollen conidia, that were stained with calcofluor white, into wild type C57/BL6 mice or mice with a targeted insertion of EGFP into the lysozyme locus (Lys-EGFP), thus harboring green neutrophils [22]. Pilot experiments had demonstrated that swollen conidia, which represent the *Aspergillus* morphotype associated with the onset of invasive growth, produced prominent NET-structures *in vitro*. After 7–10 h, mice were killed and their lungs were analysed for fluorescent cells, fungal elements as well as NETs by 2-photon microscopy (Video S3). These analyses demonstrated the formation of large fungal clusters with outgrowing hyphae and attached host cells associated with alveoli (Figure 4B and Video S8). Clearly also structures closely resembling the NETs we had observed before *in vitro* (Figure 1 and 3) were present within infected lung tissue (Figure 4B–D). The structures were especially enriched in areas with bulk associations of multiple fungal elements (Figure 4C and D) while in control animals, which only received PBS we did not observe these structures (Figure 4E). DNase digestion of these structures was possible.

**Figure 5:**
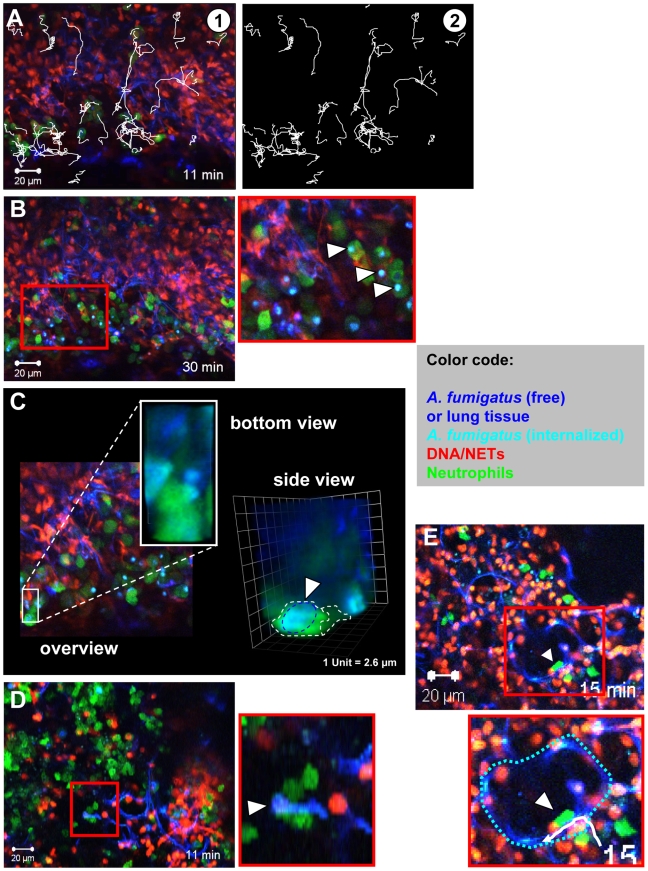
Neutrophil motility and interaction with fungal elements in living lung slices. Lys-EGFP mice were infected and acute lung slices were prepared as described in Figure 4. Subsequently, time-lapse 2-photon microscopy was used to generate movies of cells migrating in these lung slices. (A) 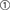 A still image of a movie showing individual neutrophils (green), DNA (red) and tissue/fungal elements (blue). Tracks of migrating cells are shown in white. 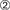 Image of the tracks alone (Video S4). (B) Many neutrophils (green) can be seen migrating within the tissue and internalising conidia in slices. The red square is shown as a magnification on the right (white arrowheads denote phagocytosis events). Tissue (dark blue) fungal elements (light blue), DNA (red, Video S5). (C) A still image from the middle of a Z-stack of an infected lung in a Lys-EGFP animal. The area boxed in white is shown enlarged from the bottom and as 3-D rendering from the side to demonstrate the internalisation of a conidium (light blue) within a neutrophil (green). See also Video S9. (D) Multiple neutrophils (green) cooperate to transport a hypha (White arrowhead, hypha is light blue. The area of the red square is shown as a magnification below.), that is too big to be engulfed, to an area with more neutrophils (Video S6). Red: DNA from nuclei and NETs. Similar events can also be observed *in vitro* (Video S10). (E) An individual neutrophil (green, arrowhead) enters the alveolar space and migrates along the alveolar surface (Dark blue structure. The white track is the migration path of the neutrophil.). The border of the alveolus is depicted with a broken blue line in the magnification of the area identified by the red square (Video S7). The images are representative of 8 individual mice that were analysed.

Neutrophils could be observed to be highly motile within these lung-slice preparations (Figure 5A and Video S4) and we measured average migration velocities of almost 10 µm/min with more than 50% of cells migrating (data not shown). Such migration parameters are very similar to values measured for neutrophils *in vivo* [23], [24], suggesting that our approach allowed the measurement of near natural neutrophil motility in vital lung tissue. Importantly, we could also observe neutrophils phagocytosing individual conidia in those living lung slices (Figure 5B, arrowheads and Video S5) leading to the localisation of conidia inside of neutrophils (Figure 5C and Video S9) and their transportation with the migrating cells over larger distances. Neutrophils could also be observed carrying swollen conidia with small hyphal segments over large distances in a collective effort (Figure 5C, arrowhead, Video S6), similar to what we had observed before *in vitro* (Figure 3 and Video S10). This was also highly reminiscent of the pattern of 2-D phagocytosis which we previously described in an *in vitro* system [4]. Sometimes individual motile neutrophils were observed migrating along the curvature of alveoli, potentially scanning the environment (Figure 5E, arrowhead, Video S7) for infection.

**Figure 6:**
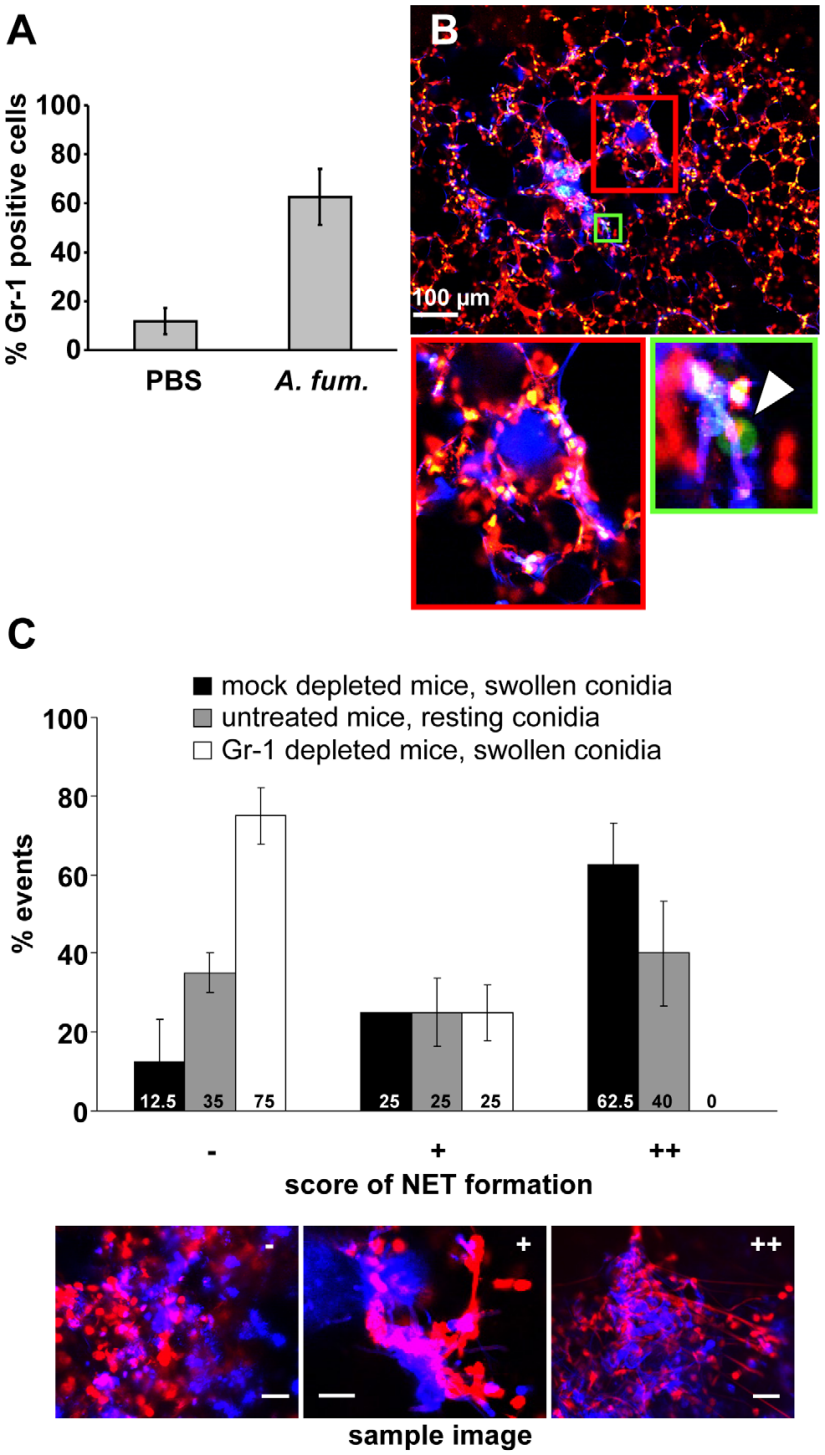
NET formation *in vivo* is dependent on the presence of newly immigrating neutrophils. (A) Mice were intratracheally injected with either swollen *A. fumigatus* conidia or PBS. 7 h after infection, the number of neutrophils in the bronchoalveolar lavage of these mice was measured by FACS. (B) Lys-EGFP mice were treated with the neutrophil depleting anti Gr-1 antibody RB6-8C5 24 h before infection with *A. fumigatus*. A lung slice of such a mouse analysed 10 h after infection shows almost no green cells (arrowhead in the magnification of the area boxed in green shows one of the very rare cells in this slice) and no NET-like structures (note only punctate red staining for DNA of nuclei) in areas of fungal masses (blue). (The area of the red square is magnified on the right) The image is representative for 3 animals that were analysed. (C) Quantification of NET formation in Gr-1- and mock-depleted mice infected with swollen conidia as well as untreated mice infected with resting conidia. Shown is a representative result of 3 independent experiments performed. For each condition 20 fungal clouds >20 µm were scored for the presence of NET structures. The 3 images are reference pictures for the type of structure scored with −/+/++.

These data strongly suggested the rapid production of NETs against an infection with *A. fumigatus in vivo*. However an important question was, whether neutrophils were required for NET formation. It could be clearly shown that neutrophils massively invaded the lung shortly after infection with *A. fumigatus* (Figure 6A). To address their importance for NET formation, we depleted neutrophils in mice by injection of anti Gr-1 monoclonal antibodies as reported [25]. 24 h later, animals were infected with *A. fumigatus* and investigated as described above. The depletion of neutrophils strongly inhibited their immigration into the lungs of infected mice. When the Gr-1 depletion was done in Lys-EGFP mice there were hardly any NET-structures detectable by staining with the DNA-specific dye Sytox Orange and no green neutrophils were patrolling the tissue (Figure 6B) despite the presence of prominent fungal clusters in the lung. A quantification of NETs in neutrophil-depleted compared to untreated mice further underscored this finding (Figure 6C). Since, however, the natural infectious particles are not swollen but rather resting conidia, we also quantified the NET formation in response to an infection with this airborne form of the fungus in untreated mice. Here, NET formation was less prominent than with swollen conidia, but still clearly detectable (Figure 6C). The almost complete lack of NET structures in neutrophil-depleted mice despite the presence of large fungal masses was prominent, thus showing for the first time a direct connection between the availability of infiltrating neutrophils in the lung and the local development of NET structures.

### The frequency of NET formation is morphotype- and strain dependent

**Figure 7:**
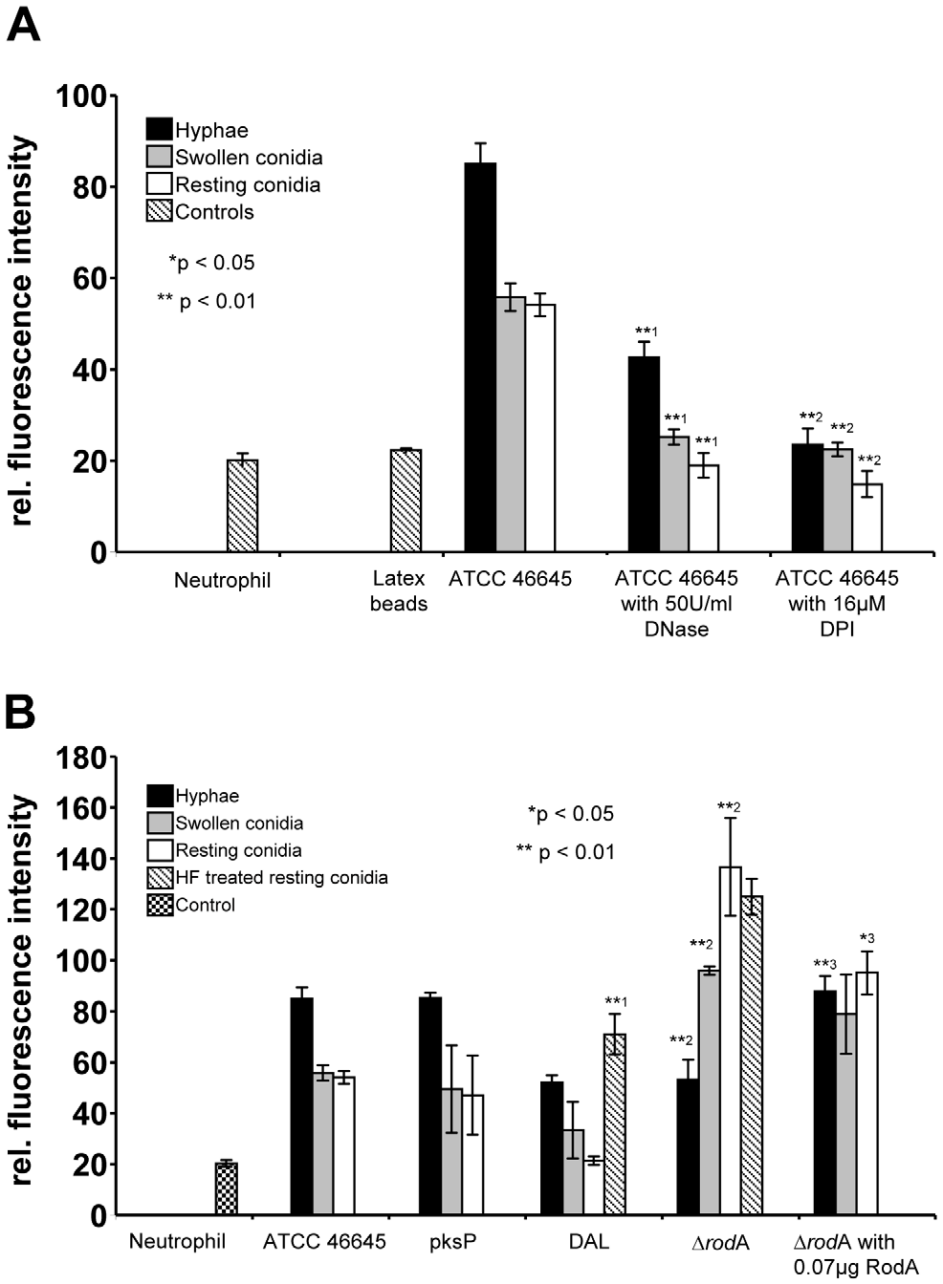
Detection of extracellular DNA by propidium iodide-staining after co-incubation of neutrophils with *Aspergillus* morphotypes. The different morphotypes of *A. fumigatus* were co-incubated for 180 min with neutrophils and the release of extracellular DNA was determined by measuring the fluorescence intensity of propidium iodide. Hyphae (black bars), swollen conidia (grey bars) and resting conidia (white bars) of *A. fumigatus* were used. (A) *A. fumigatus* ATTC 46445 wild type strain after co-incubation with human neutrophils. DNase I or DPI were added from the beginning of the co-incubation. Asterisks indicate significant differences (*p<0.05 or **p<0.01) based on Student's t-test. (^*1^) Indicates comparison of each morphotype of the wild type (ATCC 46645) strain with the DNase I treated wild type strain; and (^*2^) with the DPI treated wild type strain. (B) Analysis of different *A. fumigatus* wild type and mutant strains. In some experiments, 0.07 µg RodA protein was added to the *A. fumigatus* mutant strain Δ*rodA* just 15 min prior to the co-incubation with neutrophils. Asterisks indicate significant difference (*p<0.05 or **p<0.01) in the formation of extracellular DNA by neutrophils during (*^1^) co-incubation of HF treated resting conidia of the DAL wild type strain in comparison to untreated resting conidia as a control, during (*^2^) co-incubation with the DAL wild type strain in comparison to the mutant strain Δ*rodA, and* (*^3^) during co-incubation with the mutant strain Δ*rodA* in comparison to Δ*rodA* supplemented with the spore surface protein RodA. Only the single morphotypes were compared with each other.

To further characterise NET formation quantitatively, we analysed the DNA content of the supernatant of co-cultures of *A. fumigatus* with freshly isolated, unstimulated neutrophils using propidium iodide. Further confirming our imaging data (Figures 1 and 2), NET production in the supernatants was highest when hyphae were co-incubated with neutrophils and considerably lower with swollen and in particular resting conidia (Figure 7A). The addition of both DNase I or the NADP(H) oxidase inhibitor DPI (diphenyliodonium) led to a reduced amount of fluorescence indicating reduction in the generation of NETs (Figure 7A). Even more, the addition of DPI abolished NET formation completely which supports the finding that NET formation depends on the production of ROI [15]. The relative decrease of the number of neutrophils during the co-incubation experiments (E/T ratios of 1∶10 instead of 1∶5) with resting or swollen conidia and hyphae resulted in less NET formation, whereas ratios of 1∶1 resulted in higher fluorescence signals and thus increased NET formation (data not shown). Furthermore, NET formation also depended on the surface structure of the pathogen because latex beads did not trigger significant NET formation (Figure 7A and Figure S1E) and the measured low background fluorescence was obviously caused by neutrophils which had undergone lysis after 3 h of incubation.

To further study the ability of different fungal strains to trigger NET formation, we employed different mutant and wild type strains of *A. fumigatus* in NET-forming assays *in vitro*. As shown in Figure 7B, NET formation also depended, at least in part, on the strain analysed. NET induction triggered by the DAL wild type strain was lower than that observed with the ATCC46645 wild type strain. Interestingly, a polyketide synthase (*pksP*) mutant strain did not trigger significantly different fluorescence and thus NET production by neutrophils compared with the respective wild type strain ATCC46645. This indicated that dihydroxynaphthalene melanin, which is lacking in the *pksP* mutant, does not influence NET formation, although this cell wall component is able to suppress ROI-production in neutrophils [26], [27].

### Hydrophobin RodA influences NET formation

Nevertheless, cell wall components are the first structures of the fungal pathogen encountered by invading phagocytes and thus they should play a role in shaping the immune response against *A. fumigatus*. Since it has recently been shown that hydrophobin RodA, the major surface component of *A. fumigatus* conidia, renders them immunologically inert, thus not triggering adaptive immune responses [28], we raised the question whether RodA was able to suppress NET formation as key antifungal immune response of the innate arm of cellular immunity. Hydrophobin RodA is present on resting conidia, in reduced amounts on swollen conidia and lacking on hyphae [29]. Therefore, we analysed the Δ*rodA* mutant lacking the hydrophobin RodA surface layer of swollen and resting conidia [30]. Confirming an important role of hydrophobin RodA for this process, NET formation was significantly increased when neutrophils encountered swollen and resting *rodA* mutant conidia as compared to wild type conidia (Figure 7B). NET formation induced by resting conidia of the Δ*rodA* mutant was even stronger than the increased NET formation induced by any of the hyphal forms investigated in parallel which suggested, that hydrophobin RodA was a major factor for silencing the NET-function of neutrophils. This also indicated, that resting conidia do express a NET-inducing element that is shielded by hydrophobin RodA, as described before for the induction of adaptive immune responses [28]. NET formation was almost identical when hyphae of wild type and *rodA* mutant hyphae were compared suggesting, that a potentially strong NET-inducer that is present on resting conidia and normally shielded by RodA is lost during hyphal development. Consistently, addition of purified hydrophobin RodA to *rodA* mutant conidia reduced the NET formation (Figure 7B). Furthermore the chemical removal of the rodlet layer of DAL wild type resting conidia by hydrofluoric acid (HF) treatment, which also kills conidia, lead to a significant increase of NET formation (Figure 7B), whereas the level of NET formation stayed the same after HF treatment of resting conidia of the Δ*rodA* mutant. Obviously also dead conidia trigger NET formation and thus it appears unlikely that an actively secreted product rather than a fixed surface structure mainly activates NET formation. In addition, when RodA was already genetically removed in the Δ*rodA* mutant, HF-treatment did not further enhance NET formation by resting conidia. Taken together, these data indicate that RodA helps *Aspergillus* to evade NET induction thus constituting the first molecularly defined pathway in *A. fumigatus* for escape from this central response of neutrophils to fungal infection.

### Killing of *A. fumigatus* by neutrophils

**Figure 8:**
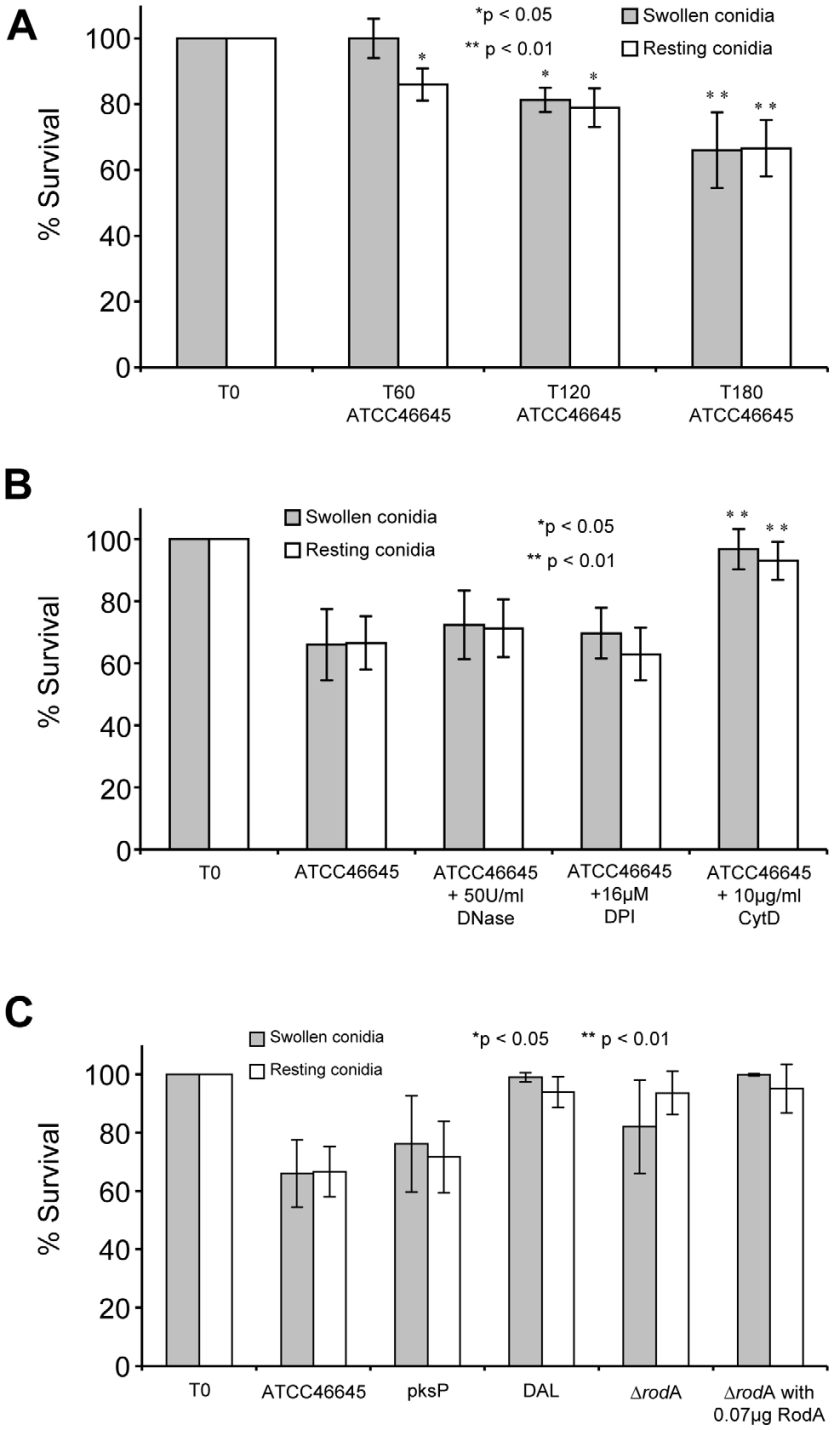
Survival of resting and swollen conidia of *A. fumigatus* strains after co-incubation with neutrophils. The number of CFU determined for conidia without co-incubation (T0) was set as 100% survival. CFUs of *A. fumigatus* conidia were determined as described in materials and methods. Survival of swollen (grey bars) and resting conidia (white bars) are depicted. (A) Survival of *A. fumigatus* strain ATCC46645 during co-incubation with neutrophils over time (T0, T60, T120 and T180). Asterisks indicate significant difference (*p<0.05 or **p<0.01) in survival in comparison to the time point T_0_ for each morphotype. (B) Survival of *A. fumigatus* strain ATCC46645 after co-incubation with neutrophils and in the presence of DNase I or DPI. Asterisks indicate significant difference (*p<0.05 or **p<0.01) in survival in comparison to the time point T_180_ after neutrophil co-incubation. The survival rate did not increase significantly by the addition of DNase I or the NAD(P)H-oxidase inhibitor DPI. Only the addition of cytochalasin D increased the survival of *A. fumigatus* conidia. (C) Analysis of the survival of the *A. fumigatus* strain *pksP* and its parental wild type strain ATCC46645 showed no significant differences in killing. Also the deletion mutant Δ*rodA* and its parental wild type strain DAL revealed no difference in killing. The addition of the conidial hydrophobin RodA (0.07 µg [w/well]) did not influence the survival of the *Aspergillus fumigatus* mutant strain Δ*rodA* and the wild type strain DAL during co-incubation with neutrophils.

Despite the clear induction of NET formation we did not observe an influence of NET formation on killing of *A. fumigatus* resting and swollen conidia *in vitro*. *A. fumigatus* conidia were co-incubated with freshly isolated, unstimulated human neutrophils and CFUs of the fungus were determined at different time points. As shown in Figure 8A, after 180 min about 35% of both swollen and resting conidia were killed. This killing rate was in the range found in previous studies, in which killing rates of around 50% of all conidia were observed after 160 min [9]. Addition of DNase I and DPI did not affect the killing of conidia (Figure 8B). Therefore, it seems unlikely that NET formation contributes to killing of conidia in this system. To elucidate whether killing can mainly be explained by phagocytosis, we added cytochalasin D, which disrupts actin filaments and thus inhibits phagocytosis, to conidia-neutrophil co-incubation experiments. Cytochalasin D effectively inhibited the killing of *A. fumigatus* conidia by naïve neutrophils (Figure 8B). So we suggest that the killing of conidia is mainly caused by phagocytosis and thus not by NET formation.

Consistently, the Δ*rodA* conidia were killed at almost the same rate as the parental wild type conidia (DAL strain), although the induction of NET formation differed significantly between the two strains. The addition of 0.07 µg hydrophobin RodA did not influence the killing of Δ*rodA* in comparison to untreated Δ*rodA* conidia (Figure 8C) significantly. Taken together, these data indicate that NET formation does not directly affect killing of conidia in this system *in vitro*.

**Figure 9:**
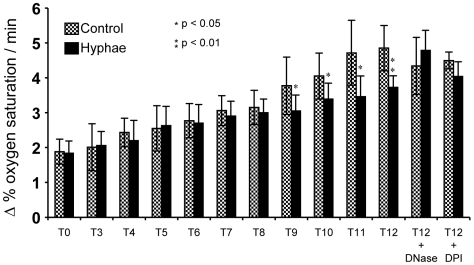
Determination of the atmospheric molecular oxygen consumption of *A. fumigatus* hyphae after co-incubation with neutrophils for 3 to 12 h. Hyphae (black bars) were co-incubated with neutrophils for different periods of time. Untreated, hyphae not co-incubated with neutrophils served as controls (stipled bars). The change of the oxygen saturation in the medium (in %) over time (h) was plotted. Asterisks indicate significant difference (*p<0.05 or **p<0.01) in the change of oxygen saturation in comparison to the control. In some experiments DNase I or DPI was added to the co-incubation and the control.

To unravel the role of NETs in killing *A. fumigatus* hyphae we measured the respiration rate of hyphae after different time periods of co-incubation with neutrophils. Since conventional CFU determination is almost impossible for the hyphal growth form of filamentous fungi, the analysis of the oxygen consumption rate served as an indirect parameter for cell viability. The first significant differences in oxygen consumption of hyphae after co-incubation with neutrophils were detected after 9 h and increased further at later time points (up to 12 h) (Figure 9) in comparison to untreated controls. The addition of DNaseI or DPI almost completely abolished the detrimental effect of the neutrophils. These findings suggest that NETs do reveal antifungal activity against fungal hyphae, which, however, occurs with a certain time lag at later stages.

## Discussion

Here, we demonstrate that both human and murine neutrophils produce neutrophil extracellular traps (NETs) in response to the human-pathogenic fungus *A. fumigatus*. Typical NET-structures which have already been described for other pathogens were observed by fluorescence and electron microscopy during co-incubation of neutrophils with *A. fumigatus* mycelium and conidia. Both fungal morphotypes were embedded in NETs consisting of smooth fibres and globular domains as first described by Brinkmann et al. [14] and others (reviewed in [16]). The DNA intercalating dye propidium iodide stained NETs strongly. EM revealed that neutrophils engulf *A. fumigatus* hyphae, a phenomenon which has also been described for *C. albicans* hyphae [21], [31]. NET formation started after 2 hours of co-incubation and increased rapidly within the next hour. A similar time span of 180–240 minutes for the release of NETs after stimulation of naïve neutrophils with *Staphylococcus aureus* was reported by Fuchs et al. [15]. Remarkably, NETs were induced relatively quickly by *A. fumigatus* conidia and mycelium in naïve neutrophils without prior stimulation, but also other eukaryotic pathogens are able to trigger NET formation *in vitro*, such as the protozoan *Leishmania* [32].

Besides experimental *in vitro* data, we provide the first direct observation of NETs or at least NET-like structures in lung tissue infected with *A. fumigatus* and show that these structures form within 3–4 hours after exposure to the first immigrating neutrophils. The recent paper by Urban et al. identified NETs in lungs fixed 24 h after infection with *C. albicans*, thus not allowing to investigate the early kinetics of this response and also precluding analysis of cell migration in the infected site. Also the role of immigrating neutrophils was not addressed in this study [21]. Our study is thus an important step forward in being the first to demonstrate the existence of NETs in *Aspergillus*-infected lungs and highlighting the importance of newly arriving neutrophils for their generation. This information is critical for a complete understanding of neutrophil defence during fungal attack. A recent study impressively demonstrated that the lack of functional NAD(P)H oxidase in neutrophils of a patient suffering from chronic granulomatous disease inhibited the production of NETs in response to *Aspergillus nidulans in vitro*. Re-introduction of a functional enzyme by gene therapy rescued the NET-phenotype *in vitro* and enabled the patient to eradicate a therapy-resistant invasive aspergillosis [19]. However, due to technical limitations the study did not directly demonstrate the existence of NETs in the infected patient lung nor could it demonstrate that the infiltration of functional neutrophils was essential for their formation. Our study closes this gap in our knowledge and provides the first direct hint to neutrophil-derived NET formation in response to *A. fumigatus* infection *in vivo*.

Our data also show the explosive release of the NET DNA, which occurs within a few seconds, while the preceding process, that prepares a neutrophil for the final NET-release, lasts up to 3 hours. The release kinetic and the fact that the structures we observe *in vitro* are highly sensitive to DNase I-mediated destruction well agree with recently published data [15] and further confirm that we were visualising true NETosis. It is interesting to note that only a subpopulation of neutrophils actually ended up producing NETs, although this was also dependent on the chosen E/T ratio. Often, a majority of cells either stayed alive or underwent normal necrotic or apoptotic cell death as detected by entry and permanent residence of nuclear dyes in cells but not the explosive release of DNA. This was despite the fact that most if not all neutrophils briefly touched or stayed in close contact with fungal elements in these assays. Also our analyses of neutrophils migrating in live lung-tissue underscored, that only a minority of neutrophils secrete NETs. Although we frequently observed NET structures closely associated with fungal masses in lung-slices, we also observed large numbers of highly motile neutrophils in between.

What ultimately decides, whether a neutrophil performs NETosis or other types of responses after contacting fungal elements, remains unclear. It is, however, conceivable, that control mechanisms exist that limit the production of NETs because external DNA, especially in the form of nucleosomes as present in the NET structures [14], [21], is potentially harmful. Nucleosomes can be taken up by DNA-specific B cells that can then make anti-nuclear-antibodies (ANAs) because they get help from T cells specific for the histone component of nucleosomes [33]. ANAs are found in many autoimmune diseases such as systemic lupus erythematosus [33], [34] and often mediate the pathologies associated with the disease. A NET-inhibiting mechanism driven by the amount of external DNA is an attractive concept. This would, however, imply that neutrophils possess a mechanism that allows them to measure the amount of external DNA, inhibiting their further production of NETs if this amount is too high. Indeed, Toll Like Receptor 9 is a well known receptor for dsDNA [35] and very recently, new receptors for intracellular DNA have been identified [36]–[38] that might serve such a function. It would thus be interesting to study animals mutant for such proteins for their ability to generate NETs.

The novel mouse model for investigating NETs and invading neutrophils in live lung tissue introduced here proved to be a very helpful approach. We can demonstrate structures in living lung tissue that closely resemble the NETs observed before *in vitro* by confocal microscopy and scanning electron microscopy. As we show, the migration parameters of cells in our experiments are in accordance with previously published data on neutrophils observed in true intravital setups in various organs [23], [24], [39] and also our own experience for neutrophil migration *in vivo*. This supports, that the tissue slice approach maintains near-natural cellular behaviour. As it is currently not foreseeable, how true intravital 2-photon microscopy deeply within the breathing lung can be technically achieved, this new approach opens a promising new avenue for the investigation of lung-associated immune responses.

Moreover, we have identified here a novel molecular mechanism by which *A. fumigatus* conidia escape neutrophil attacks via NETs. Fungal hydrophobin RodA, which very recently was identified as being important to protect conidia from recognition by the adaptive immune response [28], now also shows its potency in protecting conidia from triggering NET formation. However, the molecular mechanisms how hydrophobin RodA achieves this reduction of NET formation still remain enigmatic. Presumably, the rodlet layer hides the immunologically active protein or carbohydrate components of the cell wall. This would also explain the significantly higher induction of NETs by hyphae in comparison to resting and swollen conidia, which apparently expose fewer immunogenic molecules. By contrast, the fungal pigment DHN-melanin appears not to be involved in evading neutrophil killing. Although the *pksP* mutant possesses a smooth, modified conidial surface layer and is not able to synthesise DHN-melanin [40], it did not induce more NET formation and it was not killed at a higher rate. The surface cell wall components responsible for the induction of NETs are presently under investigation. Also the question which phagocyte receptor is involved in the triggering of NET formation remains to be answered.

In addition, we showed that ROI are important for triggering the release of NETs by *A. fumigatus*, because the specific NADP(P)H oxidase inhibitor DPI drastically reduced NET formation, as previously shown for *Staphylococcus aureus* [15]. Furthermore, DNase I disintegrated NETs as known from other studies [14]. Surprisingly, a reduced amount of NETs was not accompanied by a reduced killing rate of conidia *in vitro*. These data propose that *A. fumigatus* conidia are killed in a NET-independent fashion. This is further supported by the fact that the phagocytosis inhibitor cytochalasin D abolished conidial killing, suggesting that phagocytosis might probably by the most important antifungal mechanism for the clearance of *A. fumigatus* conidia. However, NETs revealed slightly detrimental effects on hyphal viability demonstrated by reduced respiration rates. Killing might also be mediated by antimicrobial peptides [41] but probably also by a so far unknown mechanisms. Taken together, NETs may be involved in disarming *A. fumigatus*, e.g. by binding secreted proteins and surface structures, and may prevent further spreading, but apparently do not represent the major factor for killing. These results are in marked contrast to the clear cytotoxic effect of NETs described for *C. albicans* [21]. Thus, released granular antimicrobials may not have a fungicidal, but a fungistatic effect against *A. fumigatus*. Candidates could be the fungal growth suppressing granule protein lactoferrin, which is able to sequester iron [8], [21] or the calcium binding heterodimer calprotectin, which was recently shown to be associated with NETs [21]. Clarification of these mechanisms in the future might be instrumental in elucidating the entire molecular signalling complex that leads to NET formation and fungal damage.

## Materials and Methods

### Ethics statement

All animal experiments were in compliance with the German animal protection law in a protocol approved by the Landesverwaltungsamt Sachsen-Anhalt (file number: 203.h-42502-2-881 University of Magdeburg). The ethics committee of the University Hospital Jena did not raise any concerns and approved our study (file reference 2395-10/08). All healthy voluntary donors gave written, informed consent.

### Strains and media

*Aspergillus fumigatus* wild type strains ATCC 46645 (ATCC), DAL [42] as well as the mutant strains *pksP* [26], and ΔrodA [30] were employed. The strains were cultivated in RPMI 1640 w/o glutamine (Lonza, Wuppertal, Germany) medium with 5% (v/v) heat inactivated FCS (PAA, Cölbe, Germany).

### Cultivation conditions

For microscopical analysis by both fluorescence microscopy and scanning electron microscopy (SEM) analysis *A. fumigatus* was cultivated over night in RPMI with 5% (v/v) heat inactivated FCS at 37°C on cover slips in a wet chamber. For determining colony forming units (CFUs) and the quantification of extracellular DNA, hyphae (16 h), swollen conidia (2 h) and resting conidia were incubated in 96 well plates (Brand) in 100 µl RPMI with 5% (v/v) heat inactivated FCS at 37°C.

### Isolation of neutrophils

Human neutrophils were isolated from peripheral blood of healthy donors according to the protocol of Wozniok *et al.* [43]. After a gradient centrifugation of the blood in “Polymorphprep^R^” (Axis Shield, UK) at 550×*g*, neutrophils were collected and purified by erythrocyte lysis with ACK buffer. Then, the granulocytes were washed with HBSS buffer and diluted in RPMI media with 5% (v/v) heat inactivated FCS.

### Scanning electron microscopy

Starting with 1×10^6^ conidia, *A. fumigatus* was grown on cover slips in 100 µl RPMI media with 5% (v/v) heat inactivated FCS for 16 h. To generate swollen conidia, resting conidia were preincubated in RPMI media for 2 h before. Resting conidia and swollen conidia were co-incubated with 2×10^5^ neutrophils. The cell culture/conidia mixture was incubated at 37°C. After 180 min co-incubation, a sample was drawn and washed with 0.1 M cacodylate buffer pH 7.2 (Serva, Germany) and then fixed with 2.5% (v/v) glutaraldehyde cacodylate buffer three times for 45 min. The samples were again washed with cacodylate buffer, dehydrated in a graduated ethanol series, critical-point dried (BAL-TEC CPD030, Balzer, Liechtenstein), coated (BAL-TEC SCD 005) and analysed with a Carl Zeiss SMT (Oberkochen, Germany) scanning electron microscope. Due to the fragility of the NET-structures, disturbance of the media in each step were kept to a minimum to preserve the cellular structures.

### Immunofluorescence and confocal laser scanning microscopy (CLSM)

100 µl RPMI media with 5% (v/v) heat inactivated FCS on cover slips were inoculated with 1×10^6^ *A. fumigatus* conidia. Then, 2×10^5^ neutrophils were added and the cover slips were incubated at 37°C. After different time points (0, 60, 120 and 180 min) the media were extracted and 10 µl of a solution containing 1 µg/ml propidium iodide / 100 µg/ml calcofluor white (Sigma, Deisenhofen, Germany) were added to the cover slips and inverted on a microscopic slide. Fluorescence microscopic analysis was performed with an Axiovert 200 M/LSM 5 live confocal laser scanning microscope (Carl Zeiss, Jena, Germany). Fluorescence signals were detected using a 415–480 nm band pass filter for calcofluor white and a 560–675 nm band pass filter for propidium iodide. Images were obtained using the ZEN 2008 software (Zeiss).

### Live imaging of NETosis and NET-destruction

After 3–4 h of pre-incubation in RPMI 1640 (Biochrom, Germany) supplemented with 5% (v/v) FCS at 37°C a total of 1×10^6^ swollen *A. fumigatus* conidia were stained with calcofluor white (Sigma) for 15 min at a final concentration of 50 µg/ml. These conidia were then co-incubated with 2×10^5^ freshly isolated human neutrophils in a laboratory-made microscopy chamber containing 200 µl RPMI 1640 supplemented with 5% (v/v) FCS and 10 µl of a 10 µg/ml propidium iodide solution as described before [4]. Fluorescence and cell behaviour were monitored simultaneously at 37°C at two frames per minute using an Olympus BX61 microscope with a 60×LUMPLFL W/IR(NA 0.9) lens, together with the cellˆR software (version 2.1) from Olympus Biosystems (Munich, Germany). For the DNAse assay, the co-incubation of neutrophils and *Aspergillus* was carried out in a 96 well cell culture plate for three hours followed by calcofluor white and propidium iodide staining. After this time the co-incubation was pipetted into a laboratory made microscopy chamber and immediately before start of the time lapse microscopy 10 µl of a DNase I solution ([1 U/µl] Qiagen, Germany) were added to the medium at the border of the chamber.

### Kill assay based on colony forming units

The co-incubation of 1×10^6^ swollen and resting conidia with 2×10^5^ freshly isolated human neutrophils was carried out in 100 µl RPMI in 96 well microtiter plates (Brand, Germany). When indicated, NAD(P)H oxidase inhibitor DPI (16 µM) or DNase I (100 U/ml) were added. For inhibiting phagocytosis the neutrophils were preincubated with 10 µg/ml cytochalasin D (Sigma Aldrich, Taufkirchen) for 20 min and then added to *A. fumigatus* conidia. After 180 min, 2 µl 50 U/ml DNase I were added to destroy the NET fibres. After 10 min of incubation the sample volume was adjusted to 1 ml with ice-cold water containing 0.002% (v/v) Tween 80. The samples were vortexed and diluted 1∶100 with PBS /Tween 80 (0.002% (v/v)) solution. 10 µl of the sample was plated on Sabouraud agar plates. After 24 h of incubation at 37°C, colonies were counted.

### Viability assay based on the determination of the oxygen consumption rate

Determination of the respiration rates of *A. fumigatus* hyphae were routinely performed with an oxygen monitor (YSI 5300, YSI Life Sciences, USA) equipped with polarographic Clark-type electrodes. The depletion of dissolved oxygen in RPMI medium with 5% heat inactivated FCS was measured for 10 minutes at 37°C under continuous stirring.

Samples were prepared as follows: 1×10^7^ *A. fumigatus* conidia were grown for 16 h in 3 ml RPMI with 5% heat inactivated FCS (v/v) at 37°C and 200 rpm. After centrifugation, the supernatant was discarded and 1 ml fresh RPMI with 5% heat inactivated FCS was added. The co-incubation experiment was started with 2×10^7^ fresh isolated, unstimulated neutrophils. After two different time points (from 3 to 12 h) 10 ml ice-cold water and 10 ml PBS were added, mixed for 60 s using a Vortex mixer and centrifuged for 15 min at 4000 rpm at 21°C (Centrifuge 5810R, Eppendorf, Hamburg). The pelleted mycelium was resuspended in 3 ml fresh RPMI with 5% (v/v) heat inactivated FCS and applied to the sample chamber. Pure RPMI medium was set as 100% oxygen saturation.

### Quantification of extracellular DNA

The co-incubation experiments of *A. fumigatus* conidia or mycelium with neutrophils in black 96 well plates for 3 h was carried out as described above. In some experiments, 16 µM DPI, 100 U/ml DNase I, and 0.07 µg purified RodA was added to the wells. The ratio of *A. fumigatus* hyphae or conidia to neutrophils was 5∶1. Two µg of propidium iodide were added and fluorescence was measured (excitation filter 544 nm, emission filter 612 nm, 1300 gain) in a microtiter plate reader (Fluostar optima, BMG Labtech, Germany).

### 2-Photon microscopy in infected lungs

Swelling leading to the onset of germination in conidia was carried out by a 7 h pre-incubation step in RPMI 1640 (Biochrom) supplemented with 5% FCS (v/v) at 37°C. A total of 5×10^6^ swollen *A. fumigatus* conidia were stained with calcofluor white (Sigma) for 15 min at a final concentration of 50 µg/ml. For infection these conidia were applied intratracheally into female C57/Bl.6 mice (8–10 weeks old, Harlan, Germany) resuspended in total volume of 100 µl PBS after filtration through a 70 µm cell strainer. 7–10 h later the infected animals were sacrificed by an overdose of isofluran and the lungs were filled *in situ* with prewarmed low-melting agarose (2% w/v, Promega, Germany). After solidification for 30 minutes at 4°C the right lung lobe was prepared and cut horizontally along the midline with a vibratome (752M Vibroslice, Campden Instruments, UK). The upper half of the lung was then transferred into a Petri dish filled with PBS heated to 37°C and supplemented with Sytox Orange (Invitrogen, Germany) at a final concentration of 5 µM. 2-photon microscopy was performed using a Zeiss LSM 710 NLO microscope on an upright Axio Examiner stage equipped with a 20×NA1.0 water dipping lens (Zeiss). For imaging, different areas along the dissection were scanned down to 400 µm depth using an illumination wavelength of 800 nm detecting green (530 nm) and red (580 nm) fluorescence, as well as the Second harmonic generation (SHG)-signal and the blue calcofluor fluorescence (at 400–470 nm emission) with the external non descanned detectors (NDD). SHG detects fibrillar structures such as proteins of the extracellular matrix by their emission of light at half of the wavelength used for illumination. The frame rate for movies was up to 12 fs/minute at a fixed focal depth. See also Video S3 for an explanation of the method. The movie was made based on the 3-D structure of a real mouse lung using the GNU-licensed software Blender (www.blender.org).

### Quantification of NET formation in infected lungs

To estimate the importance of neutrophils in *in vivo* NET formation animals were treated i.p. with 100 µg anti-Gr-1 antibody (clone RB6-8C5) 24 hours prior to i.t. infection with 10^8^ calcofluor white stained WT conidia. 7 hours later the infected lungs were prepared, stained with Sytox Orange (5 µM in PBS) and observed for fungal masses with a diameter ≥20 µm by 2-photon microscopy. These structures were microscopically scored for NET formation in 3 categories: (−) no NETs detectable, (+) single NET fibres attached to the fungal cloud and (++) distinct NETs surrounding the fungal material. 20 fungal clouds were checked for NET appearance per investigated lung.

### Statistics

The Student's *t*-test was used for significance testing of two groups. For the measurement of NET formation (Figure 7A) we compared the fluorescence values for hyphae of ATCC46645 with hyphae treated with DNase I as well as with DPI. In addition, the values of swollen and resting conidia were tested for significant difference. All significant differences are labeled with an asterisk (*p<0.05; **p<0.01). For the investigation of the strain-dependent difference in NET formation (Figure 7B) resting conidia of the DAL strain were compared with resting conidia after HF treatment. In all killing experiments (Figure 8) a Student's t-test was applied.

For all *in vitro* experiments blood samples of four different donors were used: two female and two male donors. For the determination of CFU five technical replicates were applied, for quantification of NET formation eight technical repetitions were used. For the quantification of respiration rates, all experiments were repeated three times.

## Supporting Information

Figure S1 NET formation by human neutrophils co-incubated with resting conidia, swollen conidia and hyphae of *A. fumigatus* at indicated time points and controls. CLSM overlay pictures showing NET formation of human neutrophils at indicated time points. Extracellular DNA was stained with propidium iodide (red), conidia and hyphae with calcofluor white (blue). Microscopic pictures were taken after 0, 60 and 120 min. Neutrophils were co-incubated with resting conidia (A), swollen conidia (B) and hyphae (C). For control only neutrophils in RPMI media were tested after 180 min (D). Also control co-incubation with latex beads showed no NET formation (E).
(2.61 MB TIF)

Video S1 Kinetic of NET formation by human neutrophils *in vitro*. Freshly isolated human neutrophils were co-incubated with conidia of *Aspergillus fumigatus*, that had before been swollen in RPMI for 150 min. Extracellular DNA was stained with propidium iodide (red), conidia and germ tubes with calcofluor white (blue-pink). Microscopy was carried out for 5 h after onset of co-incubation while single pictures were taken every 30 seconds. Indicated is a scale bar and real time in minutes. Please note the explosive release of a NET (intensive red colour around a central neutrophil) between 189–191 minutes into the experiment.
(3.59 MB MOV)

Video S2 Kinetic of NET degradation by DNase I *in vitro*. After 180 minutes of neutrophil-*Aspergillus* co-incubation a well established NET is visible. At the beginning of the movie (0 min) DNase I was added to the medium. At 7 minutes digestion of the NET started and was finished (no NET structure detectable) by 9.5 minutes into the experiment. Indicated is a scale bar and real time in minutes.
(0.84 MB MOV)

Video S3 Investigating NET formation in the murine lung. A movie showing the sequence of events leading to the formation of NETs in murine lungs and their analysis by time-lapse 2-photon microscopy *in situ*.
(4.97 MB MOV)

Video S4 Neutrophils migrate through an *Aspergillus*-infected lung slice. A Lys-EGFP mouse was infected with swollen *A. fumigatus* conidia and 7 h later the lung was prepared as described in movie 3. The sequence shows several neutrophils (green) migrating within lung tissue (blue). Red staining is from the DNA-specific dye Sytox Orange showing cell nuclei cut open by the preparation. Please note the active motility of cells within the tissue. Indicated is a scale bar and real time in minutes.
(3.99 MB MOV)

Video S5 Neutrophils phagocytose conidia of *Aspergillus fumigatus* in an infected lung slice. A Lys-EGFP mouse was treated as described for movie 4. The sequence shows highly active neutrophils (green) migrating through the lung tissue (blue) either carrying engulfed conidia (light blue) or being caught in the act of phagocytosis. Nuclei and NETs in the area are stained red (Sytox Orange). The average velocity of neutrophils in this experiment was 9.8 µm/min and 54% of cells were migrating at any time point (activity). Indicated is a scale bar and real time in minutes.
(3.62 MB MOV)

Video S6 Neutrophils transport hyphae of *Aspergillus fumigatus* within an infected lung slice. A Lys-EGFP mouse was treated as described for movie 4. The sequence shows several neutrophils (green) cooperating in the transport of a hypha (light blue) from the centre part (starting at minute 9 of the movie) to the left, where a larger accumulation of other neutrophils is located. Please also note the general accumulation of large numbers of neutrophils in a swarm-like manner into the entire area, which is a typical feature of these cells *in vivo*. The dark blue colour indicates lung tissue and red indicates DNA (Sytox Orange). Indicated is a scale bar and real time in minutes.
(3.35 MB MOV)

Video S7 Neutrophils cross epithelial borders and migrate on the alveolar surface an infected lung slice. A Lys-EGFP mouse was treated as described for movie 4. The sequence shows several neutrophils (green) migrating within the lung slice. At 12 minutes into the movie a single cell is seen (at 5 o' clock) crossing the epithelial border of an alveolus (blue) and migrating on its surface. Red shows DNA of nuclei (Sytox Orange). Indicated is a scale bar and real time in minutes.
(1.41 MB MOV)

Video S8 Neutrophils internalise conidia in living lung slices. A Lys-EGFP mouse was treated as described for movie 4. The movie shows a single neutrophil (green) with a conidium (light blue) inside of the cell. To demonstrate that the conidium is really inside, the entire cell has been reconstructed from a Z-stack and is rotated.
(7.96 MB MOV)

Video S9 Swollen hyphae germinate in the lung and induce NET formation. A wild type mouse was treated as described for Video S4. The movie shows a series of Z-slices through a large fungal mass with clearly detectable outgrowing hyphae as well as NET-structures at the periphery of the mass and the borders of alveoli. Afterwards, the whole Z-stack is 3-D rendered and rotated to demonstrate the 3-D appearance of the fungal ball relative to the lung structures. Dimensions of the stack are given at the bottom.
(4.38 MB MOV)

Video S10 Neutrophils cooperate to transport hyphal fragments into larger accumulations. Isolated neutrophils were incubated together with hyphae in the presence of the blue DNA dye Hoechst in a laboratory made incubation chamber and analysed by live cell microscopy. The movie shows how a number of free neutrophils associate with a small hyphal fragment and transport it to an area, where a larger hypha is already associated with many other neutrophils. Similar phenomena can be observed in acute lung slices. Real time is given at the bottom of the movie.
(6.55 MB MOV)
