# Supplementary figures and images for: Production of Extracellular Traps against Aspergillus fumigatus In Vitro and in Infected Lung Tissue Is Dependent on Invading Neutrophils and Influenced by Hydrophobin RodA
Source: PLoS Pathog. 2010 Apr 29;6(4):e1000873. doi: 10.1371/journal.ppat.1000873 (PMC2861696; doi:10.1371/journal.ppat.1000873)

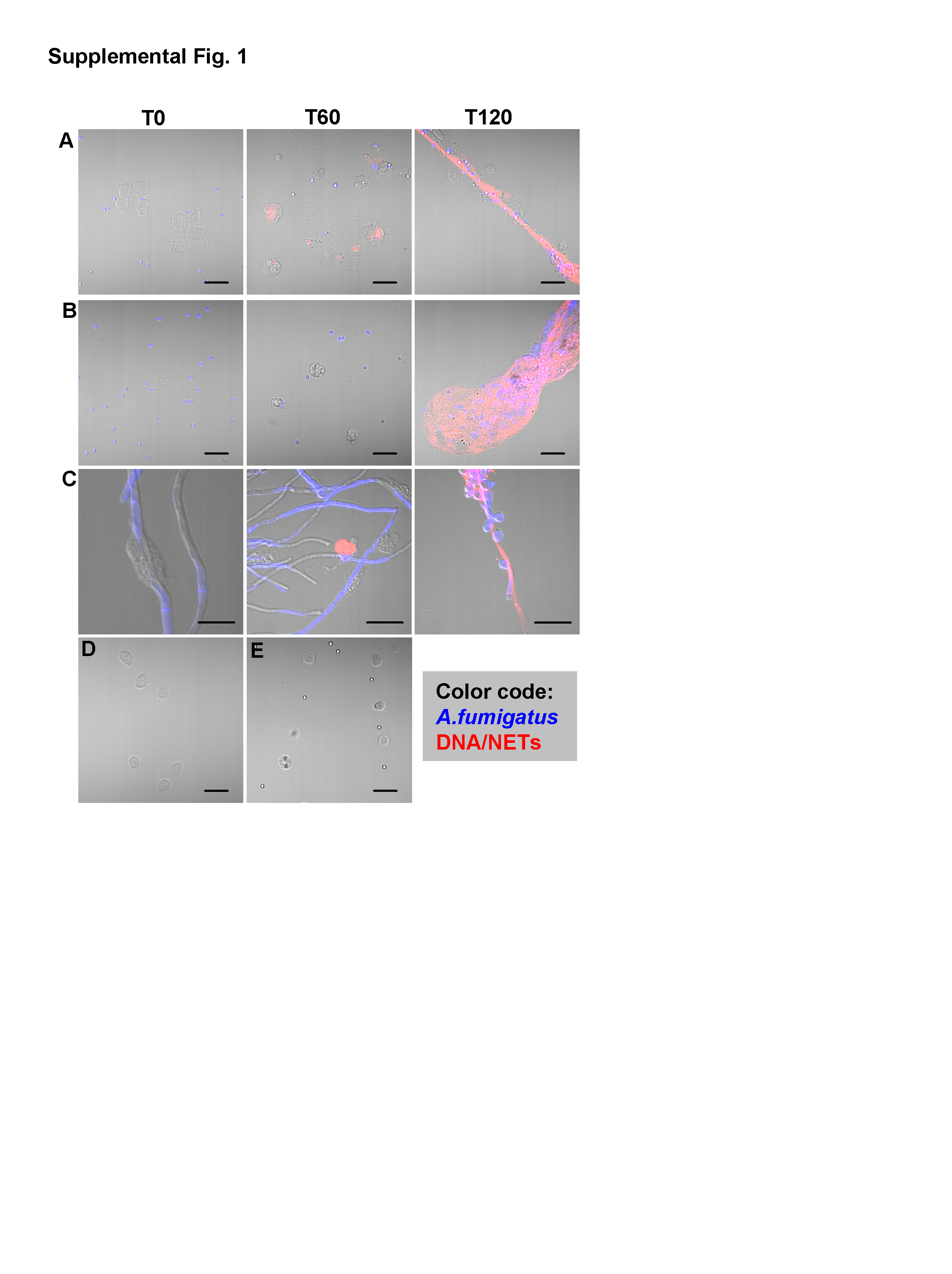

Supplement: Figure S1 — NET formation by human neutrophils co-incubated with resting conidia, swollen conidia and hyphae of A. fumigatus at indicated time points and controls. CLSM overlay pictures showing NET formation of human neutrophils at indicated time points. Extracellular DNA was stained with propidium iodide (red), conidia and hyphae with calcofluor white (blue). Microscopic pictures were taken after 0, 60 and 120 min. Neutrophils were co-incubated with resting conidia (A), swollen conidia (B) and hyphae (C). For control only neutrophils in RPMI media were tested after 180 min (D). Also control co-incubation with latex beads showed no NET formation (E). (2.61 MB TIF) [file ppat.1000873.s001.tif]
